# Risk of infection during percutaneous nephrolithotomy is the hot topic in this number of the International Brazilian Journal of Urology

**DOI:** 10.1590/S1677-5538.IBJU.2024.05.01

**Published:** 2024-08-28

**Authors:** Luciano A. Favorito

**Affiliations:** 1 Universidade do Estado do Rio de Janeiro Unidade de Pesquisa Urogenital Rio de Janeiro RJ Brasil Unidade de Pesquisa Urogenital - Universidade do Estado do Rio de Janeiro - Uerj, Rio de Janeiro, RJ, Brasil; 2 Hospital Federal da Lagoa Serviço de Urologia Rio de Janeiro RJ Brasil Serviço de Urologia, Hospital Federal da Lagoa, Rio de Janeiro, RJ, Brasil

The September-October number of *Int Braz J Urol* is the 30^nd^ under my supervision. In this number the Int Braz J Urol presents original contributions with a lot of interesting papers in different fields: Robotic Surgery, BPH, Varicocele, Testicular migration, Prostate Cancer, Prostate Biopsy, Bladder Extrophy, Endourology and Infertility. The papers came from many different countries such as Brazil, Canada, Spain, Russia, USA, Germany and China, and as usual the editor's comment highlights some of them. The editor in chief would like to highlight the following works:

Dr. Danilovic and collegues from Brazil, presented in page 561 ( 1 ) a nice study about the high-risk patients for septic shock after percutaneous nephrolithotomy, a recurrent topic in recent years in this Journal ( 2 - 4 ). The authors identified risk factors for urinary septic shock in patients who underwent percutaneous nephrolithotomy (PCNL) and concluded that patients with larger stones, positive preoperative urine culture, and a higher CCI are at risk for urinary septic shock after PCNL. These findings are of utmost importance for optimizing the perioperative care of these patients to prevent life-threatening complications.

Dr. Neto and collegues from Brazil, presented in page 530 ( 5 ) a important review about the addressing Oxidative Stress and Sperm DNA fragmentation in Varicocele-Affected Subfertile Men and concluded that the impact of varicocele grade and laterality on oxidative stress (OS) and sperm DNA fragmentation, as well as the effect of improved OS and sperm DNA fragmentation levels in pregnancy and live birth rates after varicocelectomy, are still unclear and deserve further investigation.

Dr. Favorito and Collegues from Urogential Reseacrh Unit - Brazil performed in page 519 ( 6 ) a nice review about the role of gubernaculum testis inervation in testicular migration and concluded that gubernaculum testis has important structural alterations during the testicular migration and the genitofemoral nerve and CGRP gene are of great importance in this process. The genitofemoral nerve provides motor innervation to the cremaster muscle and gubernaculum, which helps regulate the position of the testes within the scrotum.

Drs. Nikolaev and Demin from Russia performed in page 585 the cover paper of this edition ( 7 ). The authors proposed that the mobilization of the corpora under Buck's fascia, their dorsal translocation through the incisions in Buck's fascia and suturing corporal convex sides above the urethra would allow extend corporal covering of the urethra, reducing the risk of urethra-cutaneous fistula formation and concluded that the modified technique of incomplete penile disassembly applied in a homogenous group of patients with classic bladder exstrophy allows penile shaft elongation, im- proved aesthetic outcomes, preserved erections, and eliminates dorsal curvature. The technique demonstrated feasibility and reliability while maintaining positive effects on tissue circulation. The absence of urethra-cutaneous fistulae is attributed to the complete corporal covering of the urethral sutures and supports the initial hypothesis.

Dr. Morote and Collegues form Spain performed on page 595 ( 8 ) a nice study about validation of the Barcelona-MRI predictive model (BCN-MRI PM) when PI-RADS v2.1 is used with transperineal prostate biopsies and concluded that the BCN-MRI PM has been successfully validated when mpMRI was reported with the PI-RADS v2.1 and prostate biopsies were conducted via the transrectal and transperineal route.

Dr. Augustyniak and collegues from Canada, Italy, Brazil and Germany performed on page 631 ( 9 ) a nice study about the challenges, barriers and educational gaps of physicians and laboratory specialists involved in human fertility care during the COVID-19 pandemic and concluded that there is an additional need to better understand the required changes in policies and organizational processes that would facilitate access to andrology services for male infertility and specialized care, as needed.

The Editor-in-chief expects everyone to enjoy reading.
